# EGCG promotes the sensory function recovery in rats after dorsal root crush injury by upregulating KAT6A and inhibiting pyroptosis

**DOI:** 10.1515/tnsci-2022-0326

**Published:** 2023-12-14

**Authors:** Jianjun Wang, Zuer Yu, Yichun Hu, Fuyu Li, Xiaoyu Huang, Xiangyue Zhao, Yaqi Tang, Shujuan Fang, Yinjuan Tang

**Affiliations:** Department of Hepatobiliary Surgery, Affiliated Hospital of Xiangnan University, Chenzhou, Hunan, 423000, China; School of Basic Medicine, Xiangnan University, Chenzhou, Hunan, 423000, China

**Keywords:** (−)-epigallocatechin-3-gallate, dorsal root crush injury, lysine acetyltransferase 6A, pyroptosis

## Abstract

Dorsal root injury usually leads to irreversible sensory function loss and lacks effective treatments. (−)-epigallocatechin-3-gallate (EGCG) is reported to exert neuroprotective roles in the nervous systems. However, the function of EGCG in treating dorsal root injury remains unclear. Hence, we built the dorsal root crush injury (DRCI) rat model to be treated with EGCG, followed by the western blot, Enzyme-linked immunosorbent assay, and sensory behavior tests. We observed that EGCG can upregulate the Lysine acetyltransferase 6A (KAT6A) level and inhibit the pyroptosis, indicated by downregulated gasdermin-D, caspase-1, and interleukin 18 protein levels, and alleviate the neuropathic pain, indicated by the decreased paw withdraw threshold in Plantar test and decreased paw withdraw latency in von Frey test, and downregulated calcitonin gene-related peptide, nerve growth factor, and c-Fos protein levels. But EGCG cannot alleviate the neuropathic pain when the KAT6A was inhibited by CTX-0124143 and pyroptosis was activated by Miltirone. These combined results indicated that EGCG can promote the sensory function recovery in rats after DRCI via upregulating KAT6A and inhibiting pyroptosis, laying the foundation for EGCG to be a novel candidate for the treatment of dorsal root injury.

## 1 Introduction

Peripheral nerve injury is the most common and frequent cause of disability and death, and is considered one of the world’s clinical medical treatment challenges [1]. Severe peripheral nerve injury caused by trauma results in irreparable damage to nerve fibers, which gradually rupture at the distal end (known as Wallerian degeneration) and ultimately interrupt synapses with target organs, accompanied by persistent pain [2]. In the past, scientists often focused on the recovery of motor function after peripheral nerve injury, but paid relatively little attention to the recovery of sensory function after injury. Therefore, studying the methods and mechanisms of promoting sensory function recovery after peripheral nerve injury is an extremely urgent scientific issue.

The natural polyphenol compounds contained in beverages have various beneficial properties. Epigallocatechin-3-gallate (EGCG), a major active polyphenol isolated from green tea, has been extensively studied in numerous studies [3,4]. EGCG inhibits TNF- α activated NF-κB pathway and enhances nuclear factor E2 related factor 2 protein levels in macrophages [5]. EGCG alleviates staurosporine-induced cytotoxicity and apoptosis by regulating BDNF TrkB/Akt and Erk1/2 signal axes in hippocampal neurons [6]. EGCG has neuroprotective effects on the retina of ischemia-reperfusion rabbits by activating Nrf2/HO-1 [7]. Research has shown that EGCG can provide neuroprotection against brain [8], spinal cord injury [9], and sciatic nerve injury [10], alleviate neuronal cell damage caused by focal cerebral ischemia in rats [11], and promote the recovery of peripheral nerve injury function [12]. These beneficial effects are mainly attributed to the antioxidant, anti-inflammatory, and anti-apoptotic properties of free radical scavenging or EGCG [13,14,15]. Latest research indicates that EGCG can improve motor function after brachial plexus avulsion [16] and promote functional recovery after spinal cord injury [17], but there is still a lack of research on its role in sensory function. This suggests that EGCG has the potential to treat spinal dorsal root nerve injury.

Lysine acetyltransferase (KATs) catalyzes the acetylation of lysine and is a reversible protein modification involving various disease states [18]. Lysine acetyltransferase 6A (KAT6A, also known as MYST3 and MOZ) is a histone acetyltransferase of the MYST family. It has been identified for the first time in patients with acute myeloid leukemia as a mammalian KAT6A, which can control cell life processes, including inhibiting cell aging through the INK4A ARF pathway [19]. Histone acetyltransferase KAT6A/B inhibitors induce aging and prevent tumor growth [20]. More and more evidence suggests that KAT6A has been found to regulate the proliferation of normal cells [21]. KAT6A upregulates PI3K/AKT signaling through TRIM24 binding [22].

Pyroptosis is a new type of programmed cell death discovered and confirmed in recent years, which is characterized by its dependence on caspase and the release of a large number of proinflammatory factors [23]. Unlike traditional cell apoptosis, pyroptosis is defined as a special type of inflammatory necrosis characterized by cell swelling, rupture, pore formation in the cell membrane, and release of cytoplasmic contents [24]. The activation of cytoplasmic inflammatory body complexes is considered a necessary step in neuroinflammation and a key trigger factor for neuronal death [25]. During the initiation and activation of inflammasome signaling, the activated inflammasome assembles and binds to and cleaves pre caspase-1 to form an active subunit, which activates interleukin (IL)-1 β precursors, IL-18 precursors, and gasdermin D (GSDMD), further leading to inflammatory reactions and pyrophosphorylation [26]. The activation of caspase-1 is a characteristic of pyroptosis, and the activation of inflammatory bodies leads to cytokine IL-1 β and the maturity of IL-18 [27]. During central nervous system injury, inflammasomes are activated by danger signals, which subsequently induce cell apoptosis [28]. It has been confirmed that the pharmacological or genetic inhibition of inflammasome signaling or direct elimination of caspase-1 has a protective effect on neurons in brain and spinal cord injury models [29,30].

## 2 Materials and methods

### 2.1 Animals

200–220 g male Sprague Dawley rats obtained from the Guangdong Medical Laboratory Animal Center (PR China) were maintained on a 12 h light/12 h dark cycle, and was offered food and water ad libitum.

### 2.2 Dorsal root crush injury (DRCI) model procedures and groups

Animals were anesthetized by intraperitoneal injection of a mixture of methylthiazide (8 mg/kg body weight) and Ketamine (80 mg/kg body weight). Under sterile conditions, a longitudinal incision was made from the C4 to T2 spinous process (approximately 3 cm long along the midline) to separate and remove the paraspinal muscles from C4 to T2. The connective tissues and muscles were removed to expose the right spinal segment from the 4th neck (C4) to the 2nd chest (T2) level. After C5 to T1 dorsal laminectomy and dural opening, the right C5 to T1 dorsal root was exposed. Each root is crushed three times (five times each time). There are seven tweezers between dorsal root ganglion and DREZ. The Sham surgery was performed in the same way, but there was no root injury. The muscles were sutured in layers and the skin was closed with a wound clamp after completing the injury.

To investigate the neuroprotective role of EGCG after DRCI, the rats were randomly divided into 4 groups with 9 rats in each group: (A) phosphate-buffered saline (PBS), (B) PBS + EGCG, (C) PBS + EGCG + CTX-0124143 (an inhibitor of KAT6A), and (D) PBS + EGCG + Miltirone (an agonist of pyroptosis). The treatment groups were intraperitoneally injected with phosphate-buffered saline (PBS) or EGCG (10 mg/kg/day) with or without CTX-0124143/Miltirone once daily after the surgery till the day of sacrifice. The rats without DRCI treatment were used as the Sham control.

### 2.3 Sensory behavioral tests

All injured animals were tested at least once prior to injury to obtain baseline responses. From the first week post-injury, the tests were made twice a week until 6 weeks post-injury. Animals (*n* = 9 per group) were first placed in the testing room and allowed to rest quietly for half an hour. For temperature sensation testing, the responses to thermal stimuli were measured by the Plantar Test (Ugo Basile) [31]. The footpads in the rats’ forepaws were stimulated with a noxious infrared light beam which was placed below the center of the tested paw plantar. The time elapsed between the onset of infrared stimuli and the rats withdrawing their paws were recorded. The testing was repeated three times with 5 min intervals in between; the thermal stimulus cut-off time was 20 s to avoid any tissue damage.

For pressure sensation assessment, a probe connected to an electronic von Frey anesthesiometer (2390–5; Life Science Instruments) [31] was applied to the central footpads of the forepaws. The pressure was gradually increased until the rats withdrew their forepaws; the response threshold was then recorded. The testing was repeated five times with 3 min intervals in between each trail and the pressure stimulus cut-off threshold was set at 100 g to avoid tissue damage.

### 2.4 Tissue processing

At 8 weeks after the surgery, the animals were euthanized by deep anesthesia with isoflurane to be sacrificed as previously described [32,33,34,35], and then the C7 segment of the spinal cord was collected for further analyses.

### 2.5 Enzyme-linked immunosorbent assay (ELISA)

The ELISA was performed, as previously described [12,36]. The dissected tissues were homogenized in ice-cold Tris-HCl buffer (pH = 7.4, Solarbio, Beijing, China) and centrifuged at 13,000×*g* at 4°C for 10 min. ELISA was performed using commercial assay kits shown in Table 1, according to the instructions.

**Table 1:** Information on reagents

Proteins	Catalog	Company
KAT6A	JM-11280R1	Jingmei Biotechnology
GSDMD	H378	Nanjing Jiancheng Bioengineering Institute
Caspase-1	H074	Nanjing Jiancheng Bioengineering Institute
IL-18	H015-1-2	Nanjing Jiancheng Bioengineering Institute
CGRP	H217	Nanjing Jiancheng Bioengineering Institute
NGF	H043	Nanjing Jiancheng Bioengineering Institute
c-Fos	SPS-10214	Shanghai Peisen Biotechnology Institute

### 2.6 Statistics

All statistical analyses were performed by using GraphPad Prism 6 software. Data were reported as mean value ± standard deviation (SD) and analyzed using ANOVA followed by the post-hoc Bonferroni test. *P* < 0.05 was considered statistical significance.


**Ethical approval:** The research related to animals’ use has been complied with all the relevant national regulations and institutional policies for the care and use of animals. The Laboratory Animal Ethics Committee at Xiangnan University approved all experimental protocols conducted on animals.

## 3 Results

### 3.1 EGCG can upregulate the KAT6A level after DRCI

**Figure 1:**
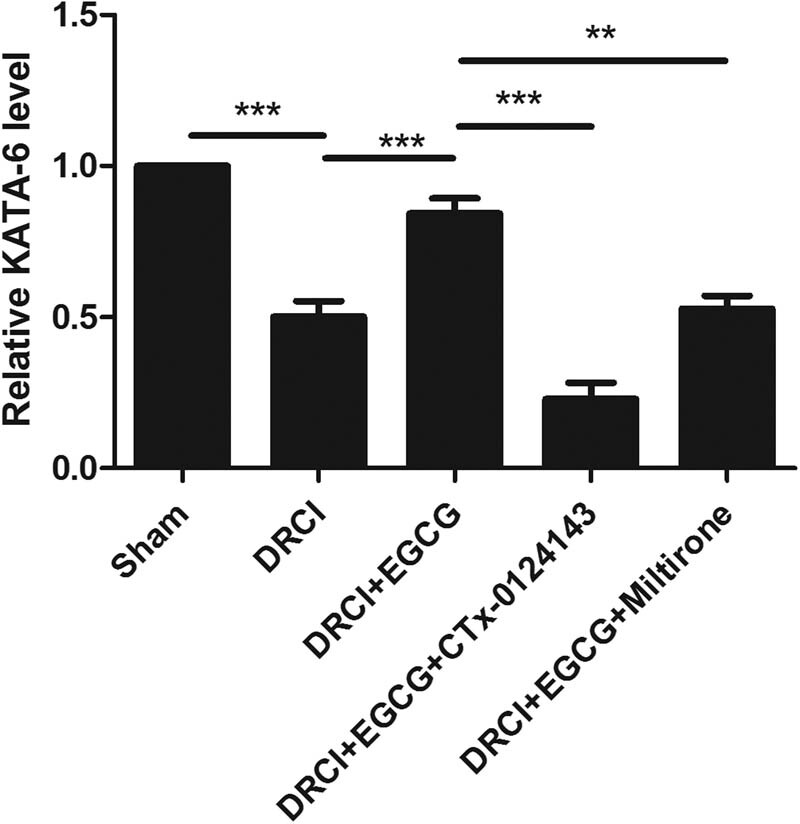
Effect of EGCG on KAT6A protein level in rats after DRCI. EGCG increased the protein levels of KAT6A (**p < 0.01, ***p < 0.001, *n* = 4, One-way ANOVA).

To evaluate the effect of EGCG on KAT6A, ELISA was performed to determine the change in KAT6A protein level. We found that the KAT6A level was downregulated after DRCI, but EGCG can upregulate the KAT6A level. In rats treated with CTX-0124143 or Miltirone, EGCG did not increase the KAT6A protein level (Figure 1).

### 3.2 EGCG can inhibit the pyroptosis after DRCI

**Figure 2:**
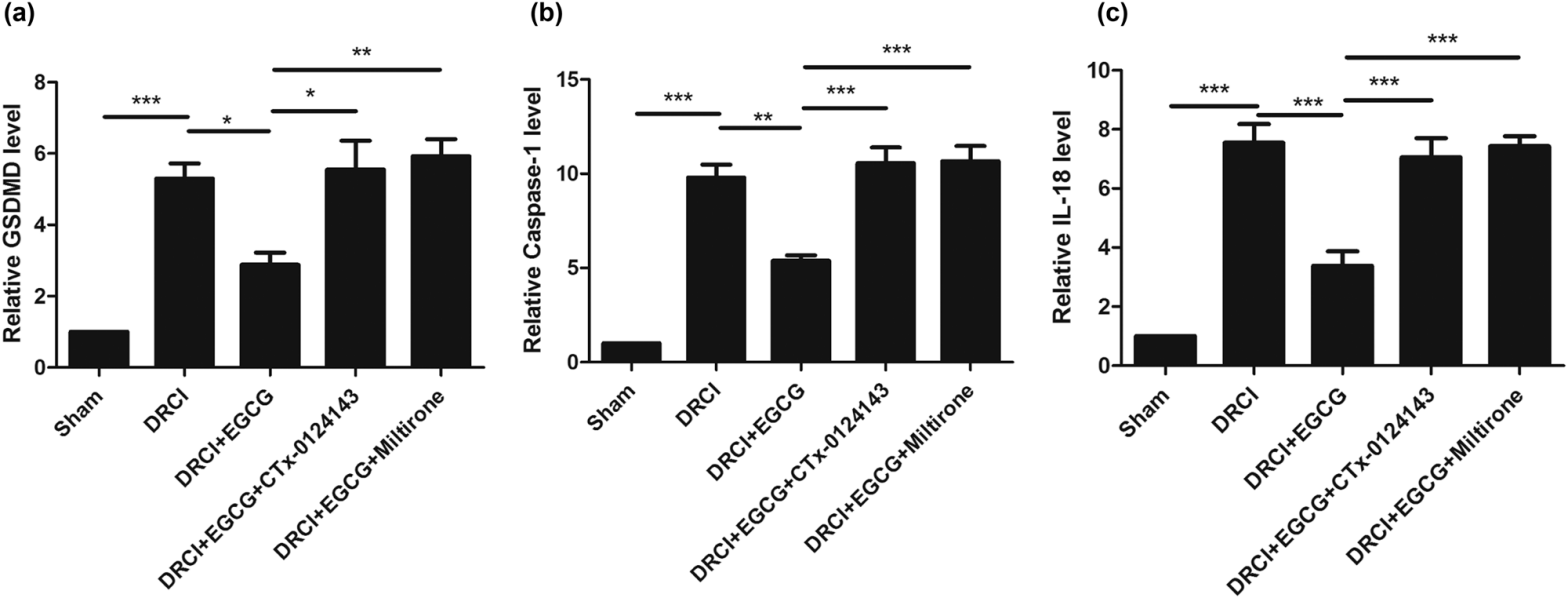
Effect of EGCG on pyroptosis in rats after DRCI. EGCG inhibited pyroptosis, as indicated by the decreased protein levels of (a) GSDMD, (b) caspase-1, and (c) IL-18 (*p < 0.05, **p < 0.01, ***p < 0.001, *n* = 4, One-way ANOVA).

To evaluate the effect of EGCG on pyroptosis, ELISA was performed to determine the change in protein levels of GSDMD, caspase-1, and IL-18. As shown in Figure 2a, we observed that, DRCI increased the GSDMD protein level, whereas EGCG decreased the GSDMD protein level. In rats treated with CTX-0124143, EGCG did not decrease the GSDMD protein level. After treating the rats with Miltirone, EGCG did not decrease the GSDMD protein level. Similar patterns for the caspase-1 and IL-18 protein levels were also observed (Figure 2b and c).

### 3.3 EGCG can alleviate the neuropathic pain after DRCI via upregulating KAT6A and inhibiting the pyroptosis

**Figure 3:**
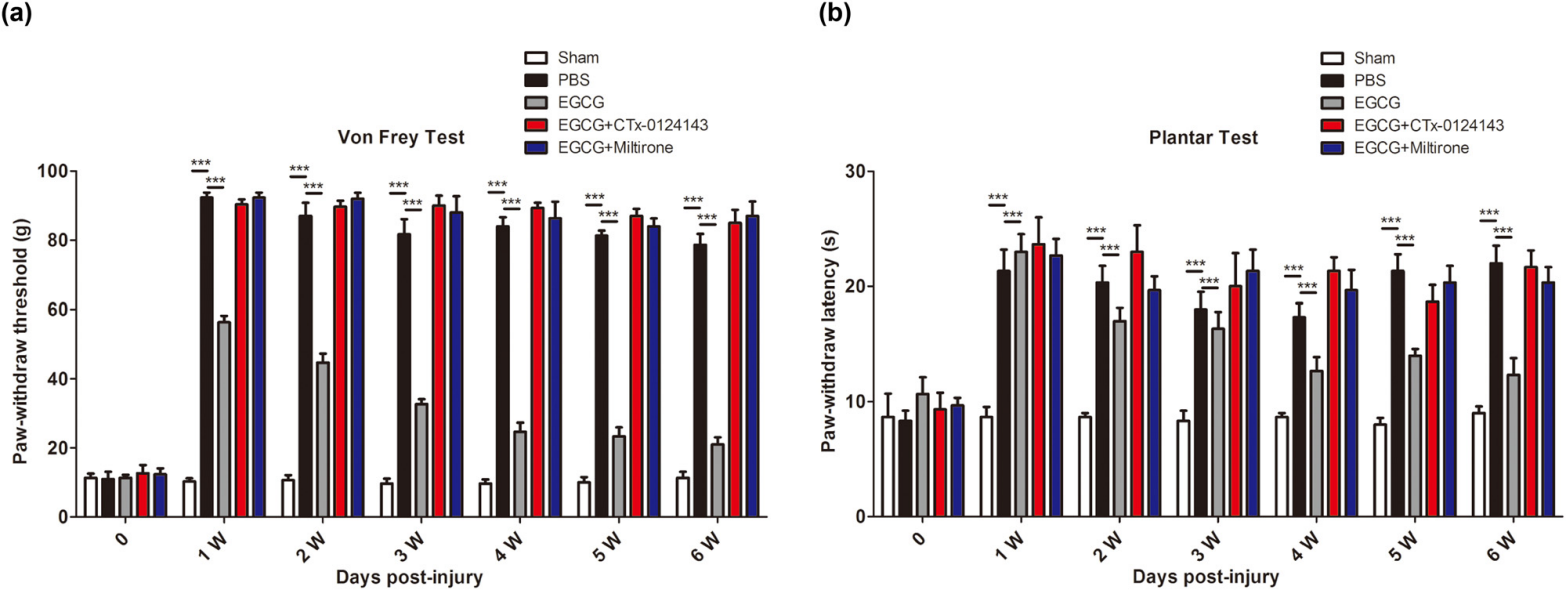
Effect of EGCG on functional recovery in rats after DRCI. EGCG upregulates KAT6A and inhibits pyroptosis to alleviate the neuropathic pain, as indicated by the decreased (a) paw withdraw threshold in von Frey test and (b) paw withdraw latency in Plantar test (***p < 0.0001, *n* = 9, Two-way ANOVA).

To evaluate the effect of EGCG on neuropathic pain, sensory behavioral tests (Plantar test and von Frey test) were performed. In Plantar test shown in Figure 3a, we observed that DRCI increased the paw withdraw threshold, whereas EGCG decreased the paw withdraw threshold. In rats treated with CTX-0124143, EGCG did not decrease the paw withdraw threshold. After treating the rats with Miltirone, EGCG did not decrease the paw withdraw threshold. A similar pattern for the paw withdraw latency in von Frey test was also observed (Figure 3b).

### 3.4 EGCG can downregulate the neuropathic pain-related proteins after DRCI via upregulating KAT6A and inhibiting the pyroptosis

**Figure 4:**
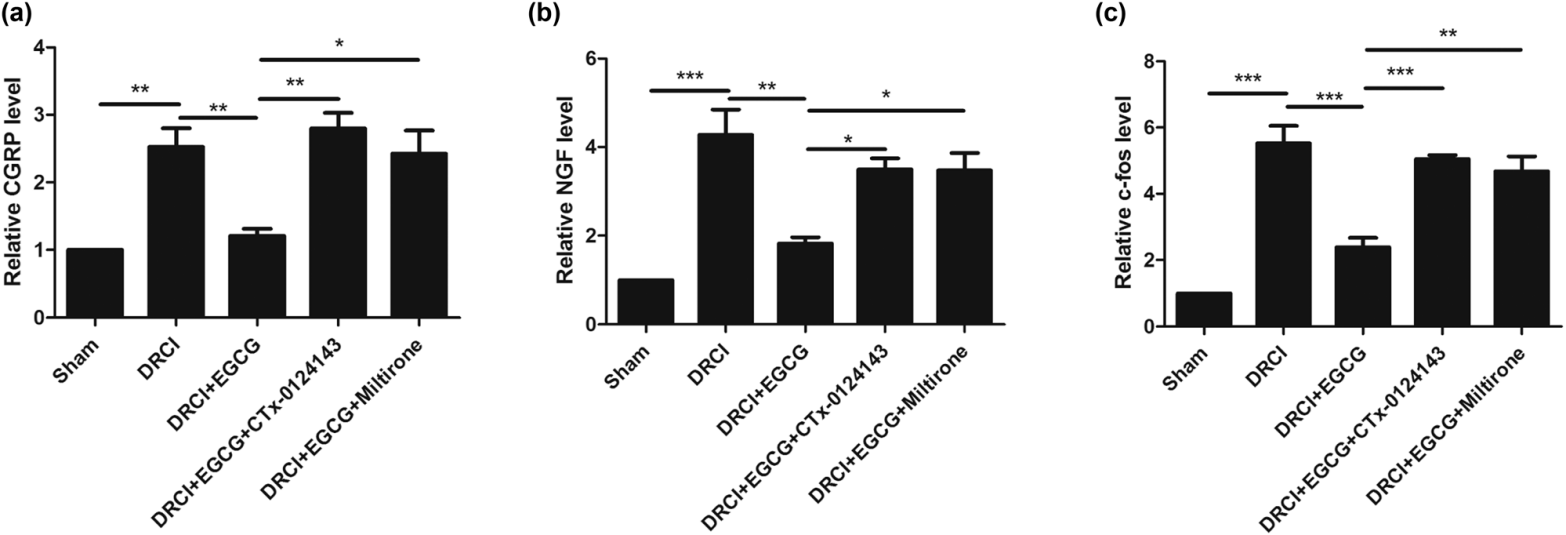
Effect of EGCG on neuropathic pain-related proteins in rats after DRCI. EGCG decreased the protein levels of (a) CGRP, (b) NGF, and (c) c-Fos (*p < 0.05, **p < 0.01, ***p < 0.001, *n* = 4, One-way ANOVA).

To evaluate the effect of EGCG on neuropathic pain-related proteins, ELISA was performed to determine the change in protein levels of calcitonin gene-related peptide (CGRP), nerve growth factor (NGF), and c-Fos. As shown in Figure 4a, we observed that, DRCI increased the CGRP protein level, whereas EGCG decreased the CGRP protein level. In rats treated with CTX-0124143, EGCG did not decrease the CGRP protein level. After treating the rats with Miltirone, EGCG did not decrease the CGRP protein level. Similar patterns for the NGF and c-Fos protein levels were also observed (Figure 4b and c).

## 4 Discussion

In the previous studies, we have revealed the effect of EGCG on the brachial plexus root avulsion [16], complete spinal cord transection [12], and intracerebral hemorrhage [37]. In the current study, we observed that EGCG can promote the sensory function recovery in rats after DRCI through upregulating KAT6A and inhibiting pyroptosis.

Assessing the neurological function is a well-known measurement to determine the degree of lesion and the therapeutic effect of strategies. In the present study, we found that EGCG can alleviate the neuropathic pain after DRCI via upregulating KAT6A and inhibiting the pyroptosis.

Neurogenic pain is considered a refractory complication after lesion [38]. Multiple nociceptive stimuli can be used to activate c-Fos, a pain biomarker, in the spinal cord [39]. NGF and CGRP were considered to exert an essential role in the molecular mechanisms of inflammatory-associated diseases and are closely involved in nerve pain [40]. In the present study, we found that EGCG can downregulate these neuropathic pain-related proteins after DRCI via upregulating KAT6A and inhibiting the pyroptosis.

Although the results seem promising, this study still has some limitations: in the current study, we mainly focused on the effect of EGCG on the sensory recovery and the underlying mechanisms, further studies are no doubt needed to pay attention to the duration of dosage of EGCG during the treatment after DRCI.

Taken together, these combined results suggest that EGCG may be a novel strategy for treating dorsal root injury.
